# Clinical and Laboratory Characteristics of Children with Chronic Idiopathic Thrombocytopenic Purpura

**DOI:** 10.3390/diagnostics15101217

**Published:** 2025-05-12

**Authors:** Milica Cekerevac, Jelena Pantovic, Marija Medovic, Nebojsa Igrutinovic, Sanja Knezevic, Bojana Markovic, Isidora Mihajlovic, Zeljko Todorovic, Tijana Maksic, Natalija Vitosevic, Suzana Zivojinovic, Jelena Cekovic Djordjevic, Tijana Prodanovic, Rasa Medovic

**Affiliations:** 1 Pediatric Clinic, University Clinical Centre Kragujevac, Zmaj Jovina 30, 34000 Kragujevac, Serbia; sanjaknez1980@yahoo.com (S.K.); bojana.kovacevic96@gmail.com (B.M.); drisidoramihajlovic@gmail.com (I.M.); tijanaveljkovic96@gmail.com (T.M.); natalijavitosevic@gmail.com (N.V.); zivojinovicsuzana@yahoo.com (S.Z.); j.cekovic86@gmail.com (J.C.D.); tijanaprodanovic86@gmail.com (T.P.); rasamedovic@gmail.com (R.M.); 2 Department of Pediatrics, Faculty of Medical Science, University of Kragujevac, Svetozara Markovica 68, 34000 Kragujevac, Serbia; 3 Center of Nuclear Medicine, University Clinical Centre Belgrade, Visegradska 26, 11000 Belgrade, Serbia; drjelena.pantovic@gmail.com; 4 Center of Dermatovenerology, University Clinical Centre Kragujevac, Zmaj Jovina 30, 34000 Kragujevac, Serbia; makastojanovic88@gmail.com; 5 Department of Dermatovenerology, Faculty of Medical Science, University of Kragujevac, Svetozara Markovica 68, 34000 Kragujevac, Serbia; 6 Endocrinology Clinic, University Clinical Centre Kragujevac, Zmaj Jovina 30, 34000 Kragujevac, Serbia; shone31094@gmail.com; 7 Department of Internal Medicine, Faculty of Medical Science, University of Kragujevac, Svetozara Markovica 68, 34000 Kragujevac, Serbia; todorovic_zeljko@hotmail.com; 8 Hematology Clinic, University Clinical Centre Kragujevac, Zmaj Jovina 30, 34000 Kragujevac, Serbia

**Keywords:** chronic idiopathic thrombocytopenic purpura, children, hematology

## Abstract

**Background/Objectives**: Chronic idiopathic thrombocytopenic purpura (chITP) is an autoimmune disease which develops in 10–30% of patients with newly diagnosed idiopathic thrombocytopenic purpura (ndITP). It is defined as thrombocytopenia which lasts longer than 12 months, with extremely diverse clinical expressions. The aim is to present the most significant clinical and laboratory characteristics of children with chITP. **Methods**: This is retrospective, observational research, which included children between 2–18 years with chITP who were treated in the Republic of Serbia for 25 years. We analyzed clinical data from personal and family medical histories and different laboratory analyses. **Results**: The total number of respondents was 152, with female predominance (F:M = 1.27:1) and mild predominance of adolescents. Of the patients, 15% were asymptomatic, but 15% had periodically life-threatening bleeding. Transfusion was not required for 70% of patients. Thirty-five percent of patients had chITP alone, and 45% had high titer levels of autoantibodies. The most frequent comorbidity was Hashimoto thyroiditis (15%). The same percentage (45%) of family members were reported with and without autoimmune diseases. Twenty-five percent of patients were resistant to initial therapy. *Helicobacter pylori* was detected in 20%, 70% had higher levels of lactate dehydrogenase (LDH), three patients had sufficient serum vitamin D levels, splenomegaly was found in 25%, and accessory spleen in 14% of patients. Around 50% of patients had a platelet count between 20–50 × 10⁹/L, and 40% below 20 × 10⁹/L. Mean platelet volume (MPV) was 10.6 ± 1.4 fL. No dysplastic changes were noted in bone marrow aspirate. Initial first-line therapy was sufficient for 45% of patients, second-line therapy was administered in 25%, splenectomy was performed in 20%, and 10% received all available treatments. **Conclusions**: The severe clinical form of pediatric chITP is accompanied by a low platelet count, the presence of autoimmune comorbidities, a positive family medical history, resistance to initial therapy, hypovitaminosis D, and rare megakaryocytes in the bone marrow.

## 1. Introduction

Idiopathic thrombocytopenic purpura (ITP) is an autoimmune disease defined by the destruction of platelets caused by the effect primary antiplatelet antibodies have against platelet glycoproteins (GPIIb/IIIa and GPIb/IX). There are three categories of ITP: (1) newly diagnosed idiopathic thrombocytopenic purpura (ndITP) (within 3 months from diagnosis), (2) persistent ITP (from 3 to 12 months) and (3) chronic idiopathic thrombocytopenic purpura (chITP) (lasting for more than 12 months) [1].

From 10 to 30% of patients with ndITP have thrombocytopenia longer than 12 months, which develops into chITP [2]. Factors at presentation that can predict which patients will develop the disease have not been defined. It is difficult to predict not only the length of achieved remission (both clinical or laboratory) in ndITP and chITP, but also which 20% of patients with chITP will spontaneously recover [2,3,4].

Previously, it was considered that chITP rarely occurs in childhood, and that children with chITP are more likely to attain spontaneous remission compared to adult patients [5]. chITP in children most commonly develops as the continuation of acute and persistent ITP. Patients’ condition deteriorates following viral infections or the intake of non-steroidal anti-rheumatic drugs [5,6]. More serious signs of hemorrhagic syndrome are shown in children, whereas higher comorbidity rates are found in adults [5,6,7]. Clinical expressions of ITP are extremely diverse, ranging from asymptomatic to life-threatening hemorrhages [1,7,8].

ITP is a diagnosis of exclusion; however, in a large cohort of nearly 4000 pediatric patients with chITP included in the registry, misdiagnosis was confirmed in approximately 3% of cases, predominantly as secondary ITP associated with underlying conditions [1,9]. It is presumed that other forms of ITP, such as those occurring in the context of systemic lupus erythematosus, antiphospholipid syndrome, connective tissue disorders, or neoplastic diseases, are similarly characterized by the binding of antiplatelet autoantibodies to specific glycoproteins or phospholipids on platelet surfaces. To the authors’ knowledge, no studies to date have investigated the emergence of other autoimmune diseases during the course of chITP; rather, existing studies have focused solely on whether an alternative diagnosis may have been masked by the initial diagnosis of chITP. Furthermore, data from the registry indicate that only 1–2% of ITP patients have a positive family history, thereby raising the question of whether ITP may have a genetic component [10].

According to recommendations by multiple authors, treatment is required for children with chITP with signs of hemorrhagic syndrome, regardless of the platelet count. Their recommendations are contradictory for patients with chITP who present with very low platelet counts (less than 10–20 × 10^9^/L) and no bleeding manifestations. On the other hand, treatment is not required for asymptomatic patients with chITP and moderate thrombocytopenia (about 50 × 10^9^/L). These patients may present with bleeding manifestations during surgical interventions, substantial injuries, or viral infections. It is considered that in patients with chITP, stable platelet counts are measured for years [1,7,8,11,12]. Approximately 60–70% of patients with chITP require some form of treatment, while others may experience spontaneous recovery. However, the factors that predict which patients will recover spontaneously have not yet been defined [1,2,3,4].

Data from the literature suggest that around 25% of patients with chITP failed to respond to standard initial first-line therapy (intravenous immunoglobulins, systemic corticosteroids) and to splenectomy [7,8,11,12]. In the last decade, there has been an increase in the clinical use of thrombopoietin receptor agonists, and preliminary results are very encouraging for both acute and chronic cases [13,14]. Various cytotoxic and immunosuppressive drugs are introduced as second-line therapy, either alone or in combination with other drugs and dosages. The drugs are experimentally administered, and their exact mechanism of action in ITP is not very clear [1,7,8,11,12]. Regardless of the number of cases, 5 to 10% of children with chITP are unresponsive to administered therapy. There is no consensus concerning the treatment of these patients which represents a serious challenge for clinicians. Increasingly, the term “refractory ITP” is being introduced to describe such cases [1,4,6,7,8,11,12,13,14,15].

The aim of this study is, based on the data from a representative number of cases, to present the most significant medical history and clinical and laboratory characteristics of children with diagnosed chITP.

## 2. Materials and Methods

### 2.1. Study Design and Participants

The study is designed as retrospective and observational research.

This research included children between the age of 2 and 18 years diagnosed with chITP who were treated in tertiary pediatric hematology centers in the Republic of Serbia for 25 years (from 1995 to 2020). Parents of children signed an informed consent before hospitalization, allowing the obtained results of laboratory analyses and data from medical records to be used for scientific purposes.

Criterion for eligibility: Diagnosed chITP according to the guidelines from the American Society of Hematology (Thrombocytopenia that lasts at least 12 months, excluding all other causes of thrombocytopenia) [1].

Criteria for ineligibility: Infants and children younger than 2 years with thrombocytopenia; children with diagnosed or suspected thrombasthenia; pregnant women younger than 18 years with chITP; children with chITP and hemostasis disorders; children with Evans syndrome; children with secondary chITP including other disorders (liver dysfunction, systemic lupus, connective tissue disorders).

Patients’ basic data were obtained from their medical history: the duration of disease, dominant bleeding manifestations, the frequency and degree of hemorrhagic syndrome according to the Bleeding Severity Score [2], presence of comorbidities in patients and other diseases in family members, the effect of therapy options in initial treatment, the frequency of transfusion of deplasmatized erythrocytes and platelet concentrates.

Regarding laboratory analyses, we measured platelet count and size, vitamin D levels, values of lactate dehydrogenase (LDH), the percentage of megakaryocytes following cytological examination of bone marrow aspirate, and the presence of different autoantibodies in patients, and tested for *Helicobacter pylori* infection. We also analyzed spleen size and diagnosed the presence of accessory spleens detected by ultrasound or during the splenectomy.

### 2.2. Statistical Data Processing

Mean values ± standard deviation are used for continuous variables, whereas categorical variables are shown in percentages. In order to determine the importance of the difference in frequencies of continuous variables, we used Student’s *t*-test, whereas a chi-squared test was used for categorical variables. The value of *p* < 0.05 was considered statistically significant.

### 2.3. Ethical Considerations

This study was conducted in accordance with the Declaration of Helsinki and received approval from the Ethics Research Committee of the University Clinical Centre Kragujevac, Republic of Serbia, with the approval code 01/22/50 dated 25 February 2022. Informed consent was obtained from the parents or legal guardians of all participating children prior to enrollment. Confidentiality and anonymity were maintained throughout the study, with all the data securely stored and accessible only to authorized personnel.

## 3. Results

The total number of children diagnosed with chITP was 152, with a mild female preponderance (F:M = 85:67, i.e., 1.27:1). The average age of our patients was 12.3 ± 3.8 years. According to age, most children were adolescents (35%), with smaller percentages of school and preschool children (around 25% in both groups) and the smallest group comprising small children. It was determined that almost 70% of all patients who had some other autoimmune disease beside chITP were females. On the other hand, 70% of patients with chITP who had positive antinuclear antibodies (ANA) or lupus anticoagulant antibodies (LAC) were exclusively males.

Table 1 shows the case distribution of children with chITP in percentages according to the severity of bleeding manifestations based on the BSS, dominant bleeding manifestations, and the need for transfusion of deplasmatized erythrocytes and platelet concentrates for the duration of disease.

From all of the observed parameters, the BSS showed correlation with platelet count in inverse proportion. It was determined that patients who received transfusions had the same ABO blood system distribution as the general population. On the other hand, 40% of patients (17 of 45) who received transfusions were Rh negative, and all the patients who received transfusions had severe bleeding manifestations (BSS 3 and 4).

**Figure 1:**
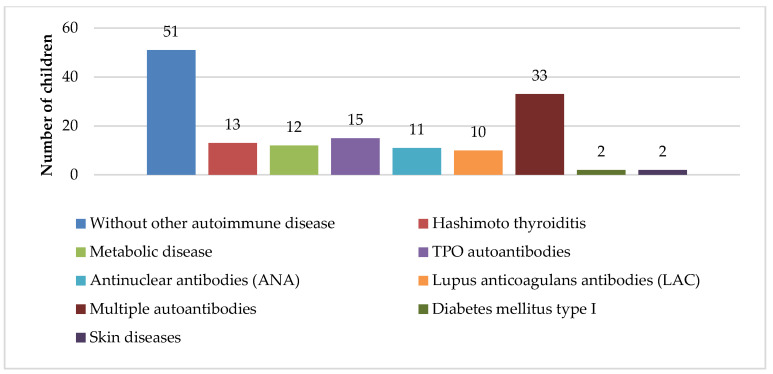
Distribution of other autoimmune diseases in children with chITP.

Figure 1 shows the distribution of other autoimmune diseases in children with chITP. Thirty-five percent of patients had chITP alone, whereas Hashimoto thyroiditis was the most frequent comorbidity found in 15% of patients, and high titer levels of autoantibodies were found in 45%, but without any clinical manifestation of the autoimmune diseases.

A statistically significant difference was noted regarding the duration of disease when the group of patients with chITP alone was compared with the group of patients that were diagnosed with an autoimmune disease. This is because a high percentage of children with autoimmune comorbidities had symptoms for more than 2 years, and most of them had severe bleeding manifestations of ITP (Table 2).

In almost the same number of family members of children with chITP, there was no report of autoimmune diseases (65 of 152 patients), or more than one family member was diagnosed with an autoimmune disease (60 of 152). Patients with autoimmune comorbidities and chITP most commonly have a positive family medical history (Table 3). Comparing the groups with positive and negative family medical histories showed that children with chITP who had relatives with autoimmune diseases were affected for more than 2 years (37 of 46) and were resistant to initial therapy (32 of 37).

Around 55% of patients with chITP had temporary response (<30 days) after initial therapy in newly diagnosed form, 25% were resistant ITP patients, whereas in 20% of patients, the response to initial therapy was good, lasting a few months or long term, mostly with complete clinical remission.

**Figure 2:**
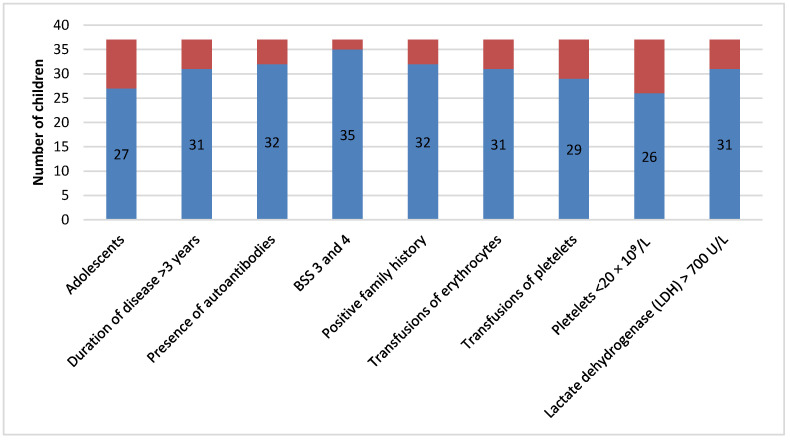
Representation of patients with chITP resistant to initial therapy (N = 37).

Figure 2 shows patients resistant to initial therapy who were most commonly adolescents. These patients presented with long duration of disease, severe bleeding, required many transfusion treatments, and had more comorbidities, a positive family medical history, lower platelet count, and higher LDH values. Furthermore, they were more resistant to corticosteroid therapy.

**Figure 3:**
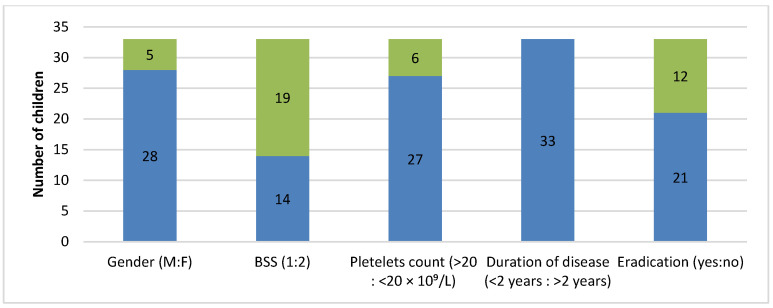
Graphic representation of children with chITP that tested positive for *Helicobacter pylori* antigen in stool samples (N = 33).

Stool samples from slightly more than 20% of patients tested positive for *Helicobacter pylori* antigen (Figure 3). It was noticeable that these patients were mostly males, asymptomatic or with mild bleeding manifestations, had a platelet count of 20–50 × 10^9^/L, and characterized by a short duration of disease (<2 years). The infection was completely eradicated in 21 patients.

Abdominal ultrasound detected splenomegaly in almost 25% of patients (37 of 152 patients), whereas the number of patients with splenomegaly was lower during the splenectomy (18 of 152). Moreover, it was noted that 14% of patients (21 of 152) had accessory spleen. It was determined that patients with splenomegaly had a platelet count lower than 20 × 10^9^/L (32 of 37) and mean platelet volume (MPV) higher than 10 fL (36 of 37). No correlation with other examined parameters was noted.

Mean platelet count at diagnosis of chITP (around 12 months) was 35.7 ± 9.6 × 10^9^/L, and MPV was 10.6 ± 1.4 fL. All the children with chITP underwent cytological examination of bone marrow aspirate, and no dysplastic changes were noted (Table 4).

When comparing the three groups of patients with different platelet counts, statistically significant differences were noted in the duration and severity of disease, number of received transfusions, and resistance to initial therapy (Table 5).

It was determined that all the patients (17 of 17) with extremely large platelets (MPV > 12 fL) who had a platelet count of <20 × 10^9^/L, belonged to the group with mild bleeding manifestations (BSS 1 and 2) and had increased production of megakaryocytes in the bone marrow. On the other hand, all the patients (23 of 23) with normal values of MPV (7–10 fL), who had a platelet count <20 × 10^9^/L, belonged to the group with severe bleeding manifestations (BSS 3 and 4) and had rare megakaryocytes in the bone marrow. This was not found in patients with mildly increased values of MPV (10–12 fL) and a platelet count >20 × 10^9^/L. Furthermore, no correlation between MPV and other examined parameters was noted.

There was no correlation between the examined parameters and normal or increased production of megakaryocytes in the bone marrow of patients with chITP. However, all patients with rare megakaryocytes in the bone marrow (34 of 34) had some autoimmune disease or were positive for autoantibodies, and many (24 of 34) had severe bleeding manifestations, received more transfusions (23 of 34), and were resistant to initial therapy (26 of 34 patients).

Almost 70% of patients had higher levels of LDH, 450–1000 U/L, and in almost a third of these, the level ranged from 700 to 1000 U/L. It was observed that extremely high LDH values (more than 700 U/L) correlated with low platelet counts <20 × 10^9^/L and resistance to initial therapy.

Only three patients with chITP had sufficient serum vitamin D levels ranging from 30 to 40 ng/mL. Around 25% of patients had hypovitaminosis D ranging from 20 to 30 ng/mL, whereas the others had vitamin D levels lower than 20 ng/mL, 17% of which had vitamin D insufficiency (<10 ng/mL). Despite the high percentage of children with chITP who had hypovitaminosis D, vitamin D insufficiency alone (<10 ng/mL) was correlated with a higher BSS (26 of 26 patients), whereas no correlation with other examined parameters was noted.

**Figure 4:**
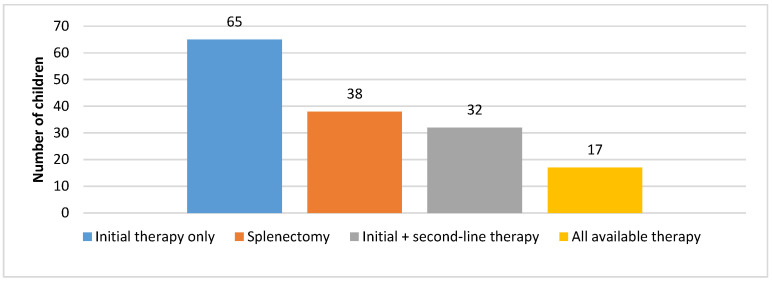
Administered therapy in children with chITP.

Figure 4 shows therapy administration in children with chITP. Approximately 45% of patients required only initial therapy in the first 12 months; afterwards, they entered a stable phase. Initial therapy and available second-line therapy were administered to 25% of patients. Around 20% of patients underwent splenectomy as the remaining treatment method, whereas 10% of patients received this treatment as part of second or third-line therapy.

## 4. Discussion

Our study of 152 cases of chITP showed that the ratio between sexes matched the data from the literature (F:M~1.3:1). Adolescents accounted for the highest percentage of affected patients (approximately 35%) [1,2,7]. One of the most reported epidemiological differences between the populations of children and adults with ITP was certainly the preponderance of females among adult patients (~2:1 ratio), while in children, the ratio was 1.3:1. This was most certainly associated with the increased incidence of autoimmune diseases in adult female patients [5,6]. Moreover, almost 70% of all patients who had autoimmune diseases were females. Official data from the literature show that disease development into chITP is very common in female children older than 10 years of age; however, numerous studies show that this development is attributed to neither the sex nor age of the patients [3,4].

After analyzing the data from patients’ medical histories, the absence of a clear cause (such as previous infections, vaccinations, insect bites or stings, etc.) is one of the commonly mentioned markers in the literature that can point to chITP in patients with ndITP. However, this may not be the rule [3,4,16]. In this cohort, at diagnosis, almost 65% of patients provided no data on a clear cause that initiated the immunological response.

In our study, approximately 15% of examined patients were asymptomatic, and 45% had skin hematomas, whereas 35% of patients had significant bleeding symptoms. Epistaxis and gingival bleeding are common accompanying symptoms and rarely dominant bleeding manifestations. To date, clinicians estimate the degree of bleeding. Although there have been attempts to establish a bleeding assessment scale [17,18], discrepancies among researchers in this field are still present. Most certainly, the percentage of asymptomatic patients with chITP ranges from 10 to 70% in different episodes [3,4,6,7,19]. In this study, we regarded patients as asymptomatic with complete clinical remission, however, with persisting thrombocytopenia after their first acute bleeding episode. Moreover, in the second year of the duration of disease, almost 45% of patients had stable platelet counts and were asymptomatic. BSS was graded 2 in approximately 55% of patients. Table 1 shows that only 15% of patients had severe, at times life-threatening, bleeding episodes (BSS of 4), which is consistent with data from the literature [3,4,6,7,19].

According to the aforementioned data, transfusion of concentrated thrombocytes or deplasmatized erythrocytes was not required for approximately 70% of patients. On the other hand, 20% of patients had to be given more than 10 transfusion treatments for the duration of disease. Transfusion was required during certain bleeding episodes for the rest of the patients. This percentage of patients is much higher than the percentages cited in the results by other researchers (between 5 and 15%) [1,3,4,6,7,19]. However, it must be considered that our study included children from Serbia with chITP in the last 25 years, in a period of national economic, political, and healthcare transition. Therefore, potential unavailability of other therapy options presumably caused an increase in the number of patients who received transfusion. It was noted that patients showed an ABO blood system distribution that was no different from that in the general population. Moreover, it should be noted that approximately 40% of patients who received transfusions were Rh negative. To date, there are no cases in which this has been documented.

An interesting difference between pediatric patients and adult chronic patients with ITP is the incidence of comorbid conditions—the presence of ≥1 comorbidity in only 5% of pediatric patients and >30% of adults at presentation, whereas after 2 years of measurement, comorbidities were present in 10–15% of pediatric patients and in 35–50% of adults [3,4]. In our study, approximately 35% of patients were diagnosed with ITP alone, whereas Hashimoto thyroiditis was diagnosed as a comorbidity in only 15% of patients. Few patients were diagnosed with hormonal or metabolic imbalance (polycystic ovary syndrome, insulin resistance, obesity), type I diabetes, vitiligo, or psoriasis. The remaining 45% of patients had high titer levels of antithyroglobulin antibodies, ANA and/or LAC antibodies, alone or in combination; however, no clinical manifestations of the autoimmune disease were noted (Figure 1). Positive test results for autoantibodies are mentioned in other studies; however, to date, their significance has not been fully understood [1,3,4,20,21,22]. While certain authors recommend monitoring and associate ANA positivity with response to therapy [19], others question whether this is truly necessary [21,22]. A very interesting result was obtained in our study: when analyzing only patients with chITP and positive for antithyroglobulin antibodies, ANA, and/or LAC antibodies, either individually or in combination, but without clinical manifestations of autoimmune disease, it was found that nearly 70% of these patients were boys (47 out of 68), a finding that, to date, has not been documented in the literature. However, due to the small number of patients included in this and other studies, more extensive research is needed. The authors intend to analyze the outcomes of this specific subgroup of chITP patients positive for autoantibodies and publish the findings in a subsequent study. In our study, a higher percentage of children with chITP and autoimmune comorbidities were affected for more than 2 years (20% with no comorbidities compared to 35% with comorbidities), and a higher percentage of patients had severe bleeding manifestations (6% compared to 40%) (Table 2). Furthermore, 50% of these patients had a positive family medical history of autoimmune diseases.

Approximately 45% of families of children with ITP reported no autoimmune diseases. However, autoimmune diseases were reported in more than one family member of the patients in almost the same percentage. Cases with a single reported disease in the family are rare, with functional disorder of the thyroid gland being the most common one. Only five patients had relatives who suffered from ITP. It is shown that a high percentage of children with chITP who had relatives with autoimmune diseases were affected for more than 2 years and resistant to initial therapy (Table 3). Several authors tried to determine attribution of genetic predispositions and the importance of autoimmune diseases in the family history for the onset of ITP and development of chITP in their studies. The authors do agree that both the onset and development of ITP can be attributed to a positive family history. However, to date, there are no consistent results regarding genetic predisposition according to ITP development [1,2,21].

Our patients with the newly diagnosed form of ITP did not go through the “wait and see” approach. Approximately 55% of the patients had a temporary response to initial therapy. Most commonly, it included laboratory, but rarely clinical, relapses that occurred 5–6 weeks following intravenous immunoglobulin treatment, lowering doses of Prednisone, or after no clinically meaningful response to pulse Dexamethasone was noted. Around 25% of patients were resistant to therapy (unachieved remissions or temporary relapse), whereas 20% had a good response to therapy that lasted for a few months or achieved complete clinical remission. Apart from the aforementioned standard therapy options for the treatment of ndITP, in the last decade, other approaches that include certain second-line therapies have emerged. However, the results obtained by researchers who examined the effects of standard therapy were similar to the results in our study [1,2,3,4,8,11,12,13,16]. Patients resistant to initial therapy were mostly adolescents who presented with longer disease duration, severe thrombocytopenia, and severe bleeding manifestations, many of whom presented with comorbidities or autoantibodies and a positive family medical history (Figure 2). Higher resistance levels were measured when corticosteroid therapy was administered rather than intravenous immunoglobulin therapy. All the aforementioned parameters can be found in the data from the literature as markers of chITP [1,3,4].

Around 20% of patients tested positive for *Helicobacter pylori* antigen detected in stool sample. To date, no mechanisms have been found that explain how *Helicobacter pylori* affects ITP pathogenesis. Recent analyses show a correlation between *Helicobacter pylori* infection and poor response to therapy in almost 50% of patients with ITP. Therefore, theory shows that eradication of the infection could contribute to the treatment of ndITP. On the other hand, eradication therapy for *Helicobacter pylori* in chITP patients is less efficient for the treatment of thrombocytopenia. However, the results are still quite contradictory [23]. In our study, the most common patients were young males who were asymptomatic or had mild bleeding manifestations, mild thrombocytopenia, and short duration of the disease. Unfortunately, the data on the number of patients who underwent esophagogastroduodenoscopy is absent. Medical history data show that eradication was completed in 21 of 33 patients, with no significant effect on the dominant disease (Figure 3). There is no clear evidence that these patients would, nonetheless, spontaneously recover or that eradication therapy and elimination of the cause of disease would contribute to recovery. Exact connections between the two entities are still unknown [23].

One-third of all platelets are stored in spleen sinusoids and function as reserve platelets. Old platelets are most commonly destroyed in the red pulp of the spleen. In the spleen, the pathophysiological mechanism of forming anti-thrombocyte antibodies occurs, which matches the location of platelet destruction in ITP patients. Thus, this organ deserves the greatest attention [8,11,12,24]. Using abdominal ultrasound, we detected splenomegaly in almost 25% of patients, with no significant effect on the outcome of disease. An occurrence of organomegaly excludes the diagnosis of ITP; however, this rule does not apply to chITP. The long-term destruction of platelets in the reticuloendothelial spleen system leads to their hyperplasia and occasionally causes organ enlargement depending on the activity of the immunological process. Other researchers confirm finding almost equal percentages of splenomegaly in their studies, especially those that opt for splenectomy as a therapy option for ITP [8,11,12,19,24]. Furthermore, it was noted that around 14% of the patients had accessory spleen, which had no significant effect on the outcome of disease.

Upon the diagnosis of chITP, the mean platelet count was around 35 × 10^9^/L, and the mean MPV was around 10.5 fL. All children with chITP underwent cytological examination of bone marrow aspirate, and no dysplastic changes were noted. Around 50% of patients had platelet counts between 20 and 50 × 10^9^/L, and 40% of patients had <20 × 10^9^/L (Table 4). A higher platelet count of 30–50 × 10^9^/L (depending on the author) at diagnosis of ITP is one of the important criteria for the onset of chITP [3,4]. On the other hand, in the course of chronic disease, around 85–90% of patients affected for more than 2 years presented with severe bleeding episodes (BSS 3 and 4), received many transfusions of blood derivatives, and had platelet counts of <20 × 10^9^/L (Table 5). Therefore, platelet count is the factor that determines the severity of bleeding manifestations in patients with chITP, as the results of other studies show [3,4,8,11,12,19,25].

An interesting phenomenon is noted regarding MPV. All patients with extremely large platelets (MPV > 12 fL) and platelet counts of <20 × 10^9^/L belong to the group of patients with mild bleeding manifestations (BSS 1 and 2) and increased production of megakaryocytes in the bone marrow. On the other hand, all patients with normal MPV (7–10 fL) and platelet counts of <20 × 10^9^/L have severe bleedings (BSS 3 and 4) and rare megakaryocytes in the bone marrow. Therefore, the presence of larger, young, reactive platelets may compensate for the reduced platelet count, as they are more metabolically and hemostatically active [26]. The relationship between MPV and bleeding is a significant topic for discussion. This can be attributed to the fact that the bone marrow gradually adapts and becomes chronically stimulated to produce younger, more reactive platelets on the one hand. On the other hand, the question arises regarding the influence and effect of autoantibodies on megakaryocytes. It is likely that in patients whose autoantibodies act solely on peripheral platelets, but not on megakaryocytes, the bone marrow remains capable of producing platelets efficiently. Conversely, in patients whose megakaryocytes are affected by autoantibodies, the production of smaller platelets occurs—platelets that are less active in hemostatic processes and, consequently, more prone to bleeding [1,20,21,22]. In such patients, bone marrow biopsies often reveal a reduced number of megakaryocytes, and these patients frequently present with ITP in combination with other autoimmune diseases [2,8,11].

Interestingly, normal or increased production of megakaryocytes in the bone marrow shows no correlation with other examined parameters. On the other hand, all patients with rare megakaryocytes in the bone marrow have some accompanying autoimmune disease or test positive for autoantibodies. Most of the patients have severe bleeding manifestations and show resistance to initial therapy. Therefore, autoantibodies, apart from their influence on platelets, can bind to megakaryocytes, inhibit their maturation, or cause their destruction. Moreover, autoantibodies can affect cytokines that are necessary for the growth and proliferation of megakaryocytes [1,2,8,11,27].

Almost 70% of patients had higher levels of LDH. It was noted that levels of LDH higher than 700 U/L correlated with a low platelet count (<20 × 10^9^/L) and resistance to initial treatment. Since LDH is an intracellular enzyme that is released by increased platelet destruction, these levels were expected. Frequent viral infections during a patient’s childhood cannot represent an adequate parameter for the assessment of disease activity since they can usually increase LDH levels, as opposed to infections in adulthood [5].

Only three patients with chITP had sufficient serum vitamin D levels, ranging from 30 to 40 ng/mL, whereas the remaining patients had lower serum vitamin D levels. The relation between these two entities has been an interesting topic in recent years. In some studies, the correlation was confirmed in more than 80%, which is similar to the results from our study. However, the evidence shows that vitamin D receptor gene polymorphism may be attributed to the disease rather than low levels of vitamin D alone. However, more extensive research should be conducted regarding this area [28]. Nevertheless, vitamin D supplementation proves to be efficient in patients with ITP.

For approximately 45% of patients, only initial therapy was required in the first 12 months; afterwards, it was confirmed (according to the data from their medical histories) that the patients entered a stable phase. An additional 25% of patients also entered a stable phase after initial therapy and available second-line therapy were administered. These patients were either without or with mild signs of hemorrhagic syndrome; however, the treatment was not required until the second year in the duration of disease. Around 20% of patients underwent splenectomy as the remaining treatment method, and all of these patients did not have any bleeding episodes after this intervention. Unfortunately, 10% of children with chITP received all available treatments and splenectomy as part of conducted therapy and periodically still have bleeding episodes (Figure 4). Most of the children from the last group are very severe cases, and the disease remains active after 5 years of treatment. This percentage is approximately similar to that cited in another study that dealt with this topic [3,4,8,11,12,19,26].

## 5. Conclusions

Most children with chITP show mild bleeding manifestations or are asymptomatic, whereas 15% have severe bleeding episodes for many years. Thirty percent of chITP patients required blood product transfusions. Platelet count is one of the crucial factors that determine the severity of bleeding manifestations. Factors that may contribute to the onset of more severe disease are other autoimmune comorbidities, a positive family medical history related to autoimmune diseases, resistance to initial therapy, lower MPV, and rare megakaryocytes in the bone marrow, present in patients with accompanying autoimmune disease or that test positive for autoantibodies. *Helicobacter pylori* infection correlates with a milder clinical presentation of ITP. Splenomegaly was detected in one-quarter of the patients, with no significant impact on disease outcome. Vitamin D supplementation is indicated in patients with chITP. Ten percent of children with chITP received all available treatment options and still experience periodic bleeding episodes, while the vast majority are either in stable remission or do not require treatment.

## Figures and Tables

**Table 1:** The assessment of bleeding manifestations in children with chronic idiopathic thrombocytopenic purpura (chITP).

Bleeding Severity Score (BSS)	N	%	Bleeding Manifestations	N	%	Platelet Concentrate Transfusion	N	%	Erythrocyte Transfusion	N	%
1	22	14.5	Asymptomatic	22	14.5	0	107	7.4	0	106	69.7
2	83	54.6	Hematoma	69	45.4	<5 treatments	14	9.2	<5 treatments	11	7.3
3	24	15.8	Mucosal Bleeding	7	4.6	<10 treatments	7	4.6	<10 treatments	9	5.9
4	23	15.1	Others	54	35.5	>10 treatments	24	15.8	>10 treatments	26	17.1

**Table 2:** Influence of autoimmune comorbidities on the duration of disease and BSS.

	chITP (51 Patients)		chITP + Autoimmune Comorbidities (101 Patients)	
Years of duration *	N	%	N	%
<2 years	41	80.4	65	64.4
<3 years	5	9.8	10	9.9
<5 years	2	3.9	6	5.9
>5 years	3	5.9	20	19.8
BSS *	N	%	N	%
1	18	35.3	4	3.9
2	30	58.8	53	52.5
3	3	5.9	21	20.8
4	0	0	23	22.8

* *p* < 0.05.

**Table 3:** Influence of autoimmune comorbidities and family medical history on chITP in children.

	chITP (N = 51)		chITP + Autoimmune Comorbidities (N = 101)	
Family medical history	N	%	N	%
Idiopathic thrombocytopenic purpura (ITP) reports in the family	0	0	5	4.9
Thyroid gland function disorder	4	7.8	10	9.9
Skin disease	0	0	1	1.0
Diabetes mellitus type I	2	3.9	3	2.9
Metabolic disorder	0	0	2	1.9
Negative family medical history	40	78.5	25	24.9
More than one family member with autoimmune disease reported	5	9.8	55	54.5

**Table 4:** Frequency of children with chITP according to platelet count, their size, and the percentage of megakaryocytes in the bone marrow.

Platelet Count (×10^9^/L)	N	%	Mean Platelet Volume (MPV) (fL)	N	%	Megakaryocytes in the Bone Marrow (%)	N	%
<10	19	12.5	<7	0	0	Rare	34	22.4
10–20	43	28.2	7–10	69	45.4	Normal	44	28.9
20–50	81	53.3	10–12	52	34.2	Increased	74	48.7
>50	9	5.9	>12	31	20.3	Dysplasia	0	0

**Table 5:** Influence of platelet count in children with chITP on examined parameters.

	Platelet Count					
	<20 × 10^9^/L (N = 62)		20–50 × 10^9^/L (N = 81)		>50 × 10^9^/L (N = 9)	
Years of duration *	N	%	N	%	N	%
<2 years	22	35.5	75	92.6	9	100
>2 years	40	64.5	6	7.4	0	0
BSS *	N	%	N	%	N	%
1 and 2	19	30.6	77	93.9	9	100
3 and 4	43	69.4	4	6.1	0	0
Transfusion treatment *	N	%	N	%	N	%
<5 treatments	33	53.2	75	92.6	9	100
>5 treatments	29	46.8	6	7.4	0	0
Therapy response *	N	%	N	%	N	%
Good response	36	58.1	70	86.4	9	100
Resistance	26	41.9	11	13.6	0	0

* *p* < 0.05.

## Data Availability

The raw data supporting the conclusions of this article will be made available by the authors upon reasonable request.

## Abbreviations

The following abbreviations are used in this manuscript:

ITP	Idiopathic thrombocytopenic purpura
ndITP	Newly diagnosed idiopathic thrombocytopenic purpura
chITP	Chronic idiopathic thrombocytopenic purpura
ANA	Antinuclear antibodies
LDH	Lactate dehydrogenase
LAC	Lupus anticoagulant antibodies
BSS	Bleeding Severity Score
MPV	Mean platelet volume
